# Redox-associated messenger RNAs identify novel prognostic values and influence the tumor immune microenvironment of lung adenocarcinoma

**DOI:** 10.3389/fgene.2023.1079035

**Published:** 2023-02-16

**Authors:** Chen Zhao, Kewei Xiong, Dong Bi, Fangrui Zhao, Yanfang Lan, Xiaorui Jin, Xiangpan Li

**Affiliations:** ^1^ Cancer Center, Renmin Hospital of Wuhan University, Wuhan, China; ^2^ School of Mathematics and Statistics, Central China Normal University, Wuhan, China

**Keywords:** tumor microenvironment, lung adenocarcinoma, immunotherapy, prognostic risk model, redox, oxidative stress

## Abstract

**Background:** An imbalance of redox homeostasis participates in tumorigenesis, proliferation, and metastasis, which results from the production of reactive oxygen species (ROS). However, the biological mechanism and prognostic significance of redox-associated messenger RNAs (ramRNAs) in lung adenocarcinoma (LUAD) still remain unclear.

**Methods:** Transcriptional profiles and clinicopathological information were retrieved from The Cancer Genome Atlas (TCGA) and Gene Expression Omnibus (GEO) of LUAD patients. A total of 31 overlapped ramRNAs were determined, and patients were separated into three subtypes by unsupervised consensus clustering. Biological functions and tumor immune-infiltrating levels were analyzed, and then, differentially expressed genes (DEGs) were identified. The TCGA cohort was divided into a training set and an internal validation set at a ratio of 6:4. Least absolute shrinkage and selection operator regression were used to compute the risk score and determine the risk cutoff in the training set. Both TCGA and GEO cohort were distinguished into a high-risk or low-risk group at the median cutoff, and then, relationships of mutation characteristics, tumor stemness, immune differences, and drug sensitivity were investigated.

**Results:** Five optimal signatures (ANLN, HLA-DQA1, RHOV, TLR2, and TYMS) were selected. Patients in the high-risk group had poorer prognosis, higher tumor mutational burden, overexpression of PD-L1, and lower immune dysfunction and exclusion score compared with the low-risk group. Cisplatin, docetaxel, and gemcitabine had significantly lower IC_50_ in the high-risk group.

**Conclusion:** This study constructed a novel predictive signature of LUAD based on redox-associated genes. Risk score based on ramRNAs served as a promising biomarker for prognosis, TME, and anti-cancer therapies of LUAD.

## 1 Introduction

Lung cancer has been the leading cause of cancer-related deaths for many years worldwide (Siegel et al., 2018). Despite knowledge gains in targeted therapies, mortality from non-small-cell lung cancer (NSCLC) remains high and has a 5-year survival rate of 16% (Santarpia et al., 2016). Lung adenocarcinoma (LUAD) is the most common histologic subtype of NSCLC patients (Barta et al., 2019); therefore, comprehensive and systematical studies to construct improved models and screen for novel biomarkers responsible for molecular diagnosis and prognostic prediction come to urgency.

Reactive oxygen species (ROS) are a cluster of short-lived molecules, which can oxidize other molecules and subsequently transition rapidly between species (Ryter et al., 2007; Kong and Chandel, 2018). In cancer cells, the production of ROS can be facilitated to initiate cancer and generate carcinogenesis (Helfinger and Schroder, 2018; Ghoneum et al., 2020). Moreover, increased ROS production mediates chemotherapy or radiotherapy responses by activating the downstream cell survival or death signaling cascades (Srinivas et al., 2019). Thus, ROS is recognized as a promoter of tumor proliferation and metastasis (Florean et al., 2019). Although redox homeostasis is critical for cell survival, ROS can also lead to cell deaths in multiple cancer types such as melanoma, pancreatic cancer, and head and neck cancer (Alexander et al., 2010; Lau et al., 2010; Herraiz et al., 2016). It is still a necessity to analyze the complex mechanism and role of redox status in cancers in depth.

Tumorigenesis is associated with not only genetic alteration of cancer cells but also the tumor microenvironment (TME), which includes the extracellular matrix, blood vessels, oxygen, and inflammatory cells (Khramtsov, 2018). Increasing evidence suggests that the TME enables cancer cells to respond to chemoradiotherapy and is pivotal to cancer diagnosis and therapy (Wang et al., 2018). Redox homeostasis also participates in the TME. Some antioxidants, such as Trx, TrxR, and NADPH, mediate distinct intracellular processes that induce oxidative stress and regulate the redox cellular microenvironment (Policastro et al., 2013). Normalizing the TME redox parameters may decrease the selection pressure for malignant phenotypes, therefore providing a tool for TME-targeted anticancer therapy (Khramtsov and Gillies, 2014). However, few studies investigated the patterns and characteristics of redox in the TME of LUAD.

This study identified prognostic and differentially expressed redox-associated genes based on the data downloaded from online cohorts. Subsequently, active and inhibited biological functions and pathways were determined. The immune-infiltrating levels and differentially expressed genes (DEGs) were also investigated. To examine the effectiveness of the model, the nomogram, survival analysis, independent prognostic analysis, TME, and drug sensitivity with the risk were explored and validated in an external cohort.

## 2 Materials and methods

### 2.1 Data collection and processing

The list of redox-associated mRNAs (ramRNAs) was extracted from 20 gene sets of the Molecular Signatures Database (MSigDB, http://www.gsea-msigdb.org/gsea/index.jsp), an integrated web server providing annotated gene sets of distinct species. The transcriptional profile of LUAD patients was downloaded in The Cancer Genome Atlas (TCGA, https://portal.gdc.cancer.gov/) including 59 normal tissues and 535 tumor tissues along with the corresponding clinicopathological phenotypes of patients with cancer single-nucleotide variant (SNV) information. The RNA sequencing data were formatted in transcripts per kilobase million and processed by log2 transformation. Since the prognostic information of CPTAC-3 is not available, we excluded the cohort from our study. Another independent cohort (GSE36471) of Gene Expression Omnibus (GEO, https://www.ncbi.nlm.nih.gov/geo/) was chosen to validate the results externally. The rationale of selecting this cohort included the two reasons. First, the follow-up information can be obtained, which can be used for survival analysis. Second, the overlapped genes sequenced in this dataset with TCGA contained the candidates responsible for the redox signature. Statistical and bioinformatics analyses in this study were conducted using the R program (version 3.6.3 and 4.0.5).

### 2.2 Unsupervised clustering to identify the novel subtypes of LUAD

A univariate Cox proportional hazard regression was performed to screen for survival-related ramRNAs in LUAD with the threshold of *p* < 0.05. The DEGs between normal and tumor tissues were identified with |log(Fold Change) (log FC)|>1.5 and *p* < 0.01. Then, the overlapped ramRNAs were included in K-means consensus clustering to classify LUAD patients into different subtypes based on their expression levels by using the ConsensusClusterPlus package.

### 2.3 Clinical and biological function characteristics of clustering subtypes

Kaplan–Meier (KM) analysis was conducted to estimate the statistical differences of survival outcomes among the clustering subtypes. To assess relationships between clustering and tumor purity, the “Estimation of Stromal and Immune cells in Malignant Tumors using Expression data” (ESTIMATE) algorithm (Yoshihara et al., 2013) and Wilcoxon rank-sum test were carried out to predict the differences among clustering subtypes and immune and stromal components in LUAD. This study also investigated the distribution of clinical phenotypes including the follow-up status, age, gender, pathologic stage, T stage, N stage, and tumor status in different subtypes by the chi-squared test. The subtle biological pathways were identified between each of the two clustering subtypes by gene set variation analysis (GSVA) (Hanzelmann et al., 2013) based on “c2. cp.kegg.v7.4. symbols.gmt” and “c2. cp.reactome.v7.4. symbols” gene sets retrieved from MSigDB for Kyoto Encyclopedia of Genes and Genomes (KEGG) and RACTOME enrichment, respectively. In addition, tumor immune-infiltrating levels of 28 cell types among the clustering subtypes were evaluated by single-sample Gene Set Enrichment Analysis (ssGSEA) (Barbie et al., 2009) and the Kruskal–Wallis (KW) test.

### 2.4 Determination of optimal redox-associated signatures for LUAD prognosis

The overlapped DEGs between each of the two subtypes with |log FC|>0.5 and *p* < 0.001 were included in the univariate Cox model. Significant ramRNAs were used to perform further dimension reduction through a weighted random forest and sliding window sequential forward feature selection by curtailing out-of-bag (OOB) error rate (Zhang et al., 2019) in the ranger and randomForest packages. Then, the TCGA cohort was divided into a training set and a validation set randomly at a ratio of 6:4. To validate the randomness, Pearson’s chi-squared test was performed with clinicopathological information (Bao et al., 2020). LASSO Cox regression was used to screen for optimal redox-associated signatures based on the results of feature selection with the training set. The risk score was formulated as follows:

$$\begin{array}{c} Risk\;score={\left[\begin{array}{cccc} \alpha_{1} & \alpha_{2} & \ldots & \alpha_{n} \end{array}\right]}_{1\times n}\times {\left[\begin{array}{cccc} \beta_{11} & \beta_{12} & \ldots & \beta_{1m}\\ \beta_{21} & \beta_{22} & \ldots & \beta_{2m}\\ \vdots & \vdots & \ddots & \vdots \\ \beta_{n1} & \beta_{n2} & \ldots & \beta_{nm} \end{array}\right]}_{n\times m} \end{array},$$ (1)

where the row vector represents the coefficient of the n signatures, and the matrix represents the corresponding expression levels of m samples. Patients from both TCGA cohort and GEO cohort were distinguished into a high-risk and a low-risk group at the median cutoff of risk score in the training set.

### 2.5 Model validation and combined diagnosis estimation

A log-rank test was taken to analyze the survival differences between the high- and low-risk groups in the training set, validation set, whole TCGA cohort, and GEO cohort. Moreover, the survival outcomes were compared between the high- and low-expression groups of patients classified at the optimal cutoff of transcriptional expression in each ramRNA signature. Receiver operating characteristic (ROC) curves and AUC values of 1-, 2-, and 3-year survival probability were adopted to reveal the accuracy and sensitivity of the prediction. The relationships between the survival status and risk score were investigated with the strategy of statistical difference measured by the Wilcoxon rank-sum test. Principal component analysis (PCA) was performed to visualize the discrimination of the model. Independent prognostic factors were determined by a univariate and a multivariate Cox proportional hazard regression with the clinicopathological phenotype information in the training set, validation set, and TCGA cohort. In addition, a nomogram model was developed based on the TCGA phenotype information and validated by calibration and concordance index (C-index). Decision curve analysis (DCA) was conducted to compare the net benefit between the risk score and traditional staging system.

### 2.6 Relationships of mutation characteristics and tumor stemness

The top 20 mutated genes in the low- and high-risk groups were analyzed, respectively. Furthermore, the correlation between the risk and tumor mutational burden (TMB) of each sample was estimated by Spearman’s correlation coefficients. Patients with TMB information were divided into a low-TMB and a high-TMB group at the optimal cutoff for survival. Afterward, risk and TMB groups were integrated as four types of combinations: L-Risk + L-TMB, L-Risk + H-TMB, H-Risk + L-TMB, and H-Risk + H-TMB. The survival differences among the re-constructed subtypes were tested by the KM method. Stemness indices calculated by machine learning were recognized as a novel potential biomarker in anti-cancer therapies recently (Malta et al., 2018). In the present study, the relationship between the risk and RNA stemness score (RNAss) or DNA stemness score (DNAss) obtained from the UCSC Xena website (http://xena.ucsc.edu/) was examined. Moreover, GSEA was employed to screen for a significantly enriched biological process in the low- and high-risk groups.

### 2.7 Exploration of the TME and immunotherapies with risk

To explore the immune differences between the low- and high-risk groups, the KW test was used to investigate the varieties of risk scores among multiple immunophenotypes, including wound healing (C1), IFN-gamma dominant (C2), inflammatory (C3), lymphocyte depleted (C4), and TGF-beta dominant (C6). The information of tumor immune-infiltrating levels estimated by TIMER, CIBERSORT, CIBERSORT-ABS, QUANTISEQ, MCPCOUNTER, XCELL, and EPIC was acquired from the TIMER database (http://timer.cistrome.org/). The immune-infiltrating differences and PD-L1 expression levels between risk subtypes were evaluated by the Wilcoxon rank-sum test. Furthermore, immune dysfunction and exclusion of immune escape mechanisms in LUAD according to Tumor Immune Dysfunction and Exclusion (TIDE, http://tide.dfci.harvard.edu/) were explored along with risk groups.

### 2.8 Associations of drug sensitivity and risk

The pRRophetic package (Geeleher et al., 2014) was adopted to predict the chemosensitivity measured by half maximal inhibitory concentration (IC_50_) based on transcriptional expression profiles between the low- and high-risk groups.

## 3 Results

### 3.1 Clustering subtypes based on prognostic ramRNA expression levels

**FIGURE 1:**
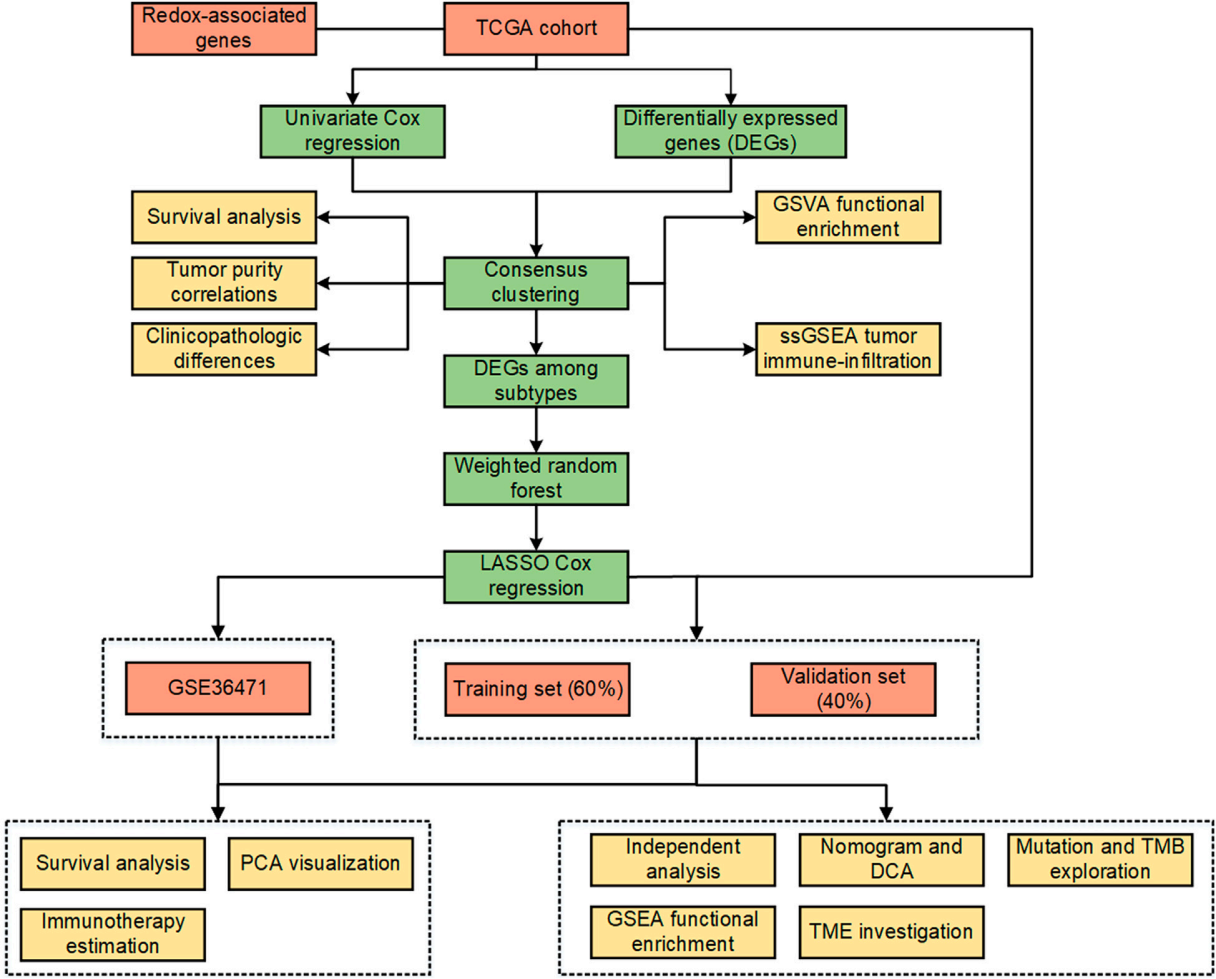
Workflow of the study.

**FIGURE 2:**
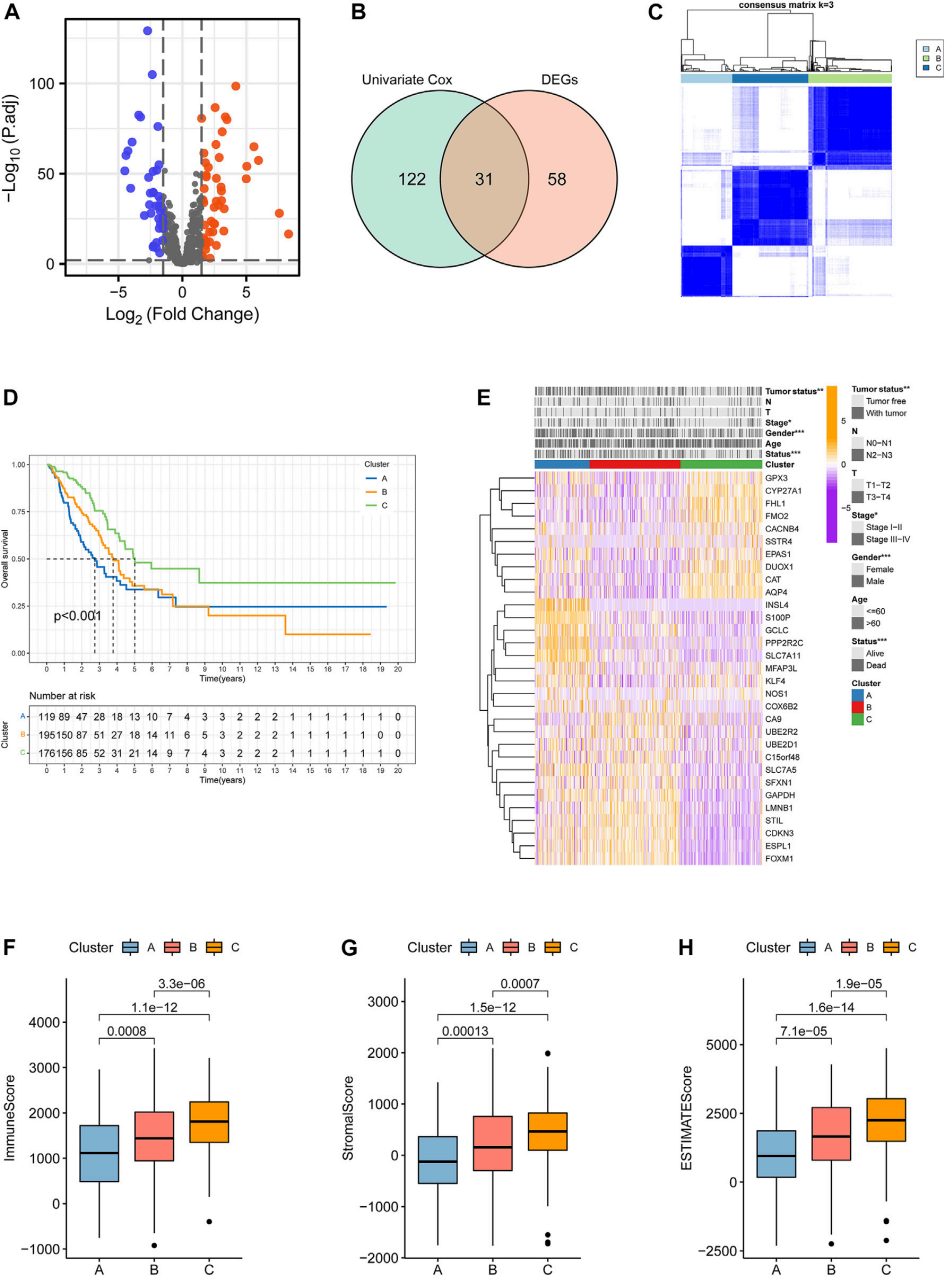
Identification of clustering subtypes based on prognostic differentially expressed ramRNAs. **(A)** Volcano plot with the thresholds of *p* < 0.01 and |log(Fold Change)|>1.5. Blue points represented downregulated ramRNAs and red points represented upregulated ramRNAs in the tumor tissues compared with normal tissues. Gray points represented non-DEGs. **(B)** The 31 overlapped ramRNAs from univariate Cox regression and DEGs are shown by a Venn diagram. **(C)** Consensus matrix with 3 clustering subtypes. **(D)** Kaplan–Meier analysis and the log-rank test showed the significant difference among Clusters A–C. Black dotted lines represented the median survival of each clustering. **(E)** TheHeatmap illustrated the expression levels of 31 DEGs and clinicopathological variable distributions among the three clustering subtypes (***p < 0.001, **p < 0.01, and *p < 0.05). Orange indicates three high-expression levels, while purple indicates a low level. **(F–H)** Differences in immune, stromal, and ESTIMATE scores among the subtypes. ramRNA: redox-associated messenger RNA and DEG: differentially expressed gene.

The workflow of the present study is shown in Figure 1. A total of 153 ramRNAs were examined by univariate Cox proportional hazard regression to identify the prognostic targets (Supplementary Table S1). A total of 89 DEGs including 39 downregulated genes and 50 upregulated genes compared within normal and tumor tissues were determined (Figure 2A, Supplementary Table S2). Then, 31 aggregated prognostic differentially expressed ramRNAs were obtained (Figure 2B). Unsupervised consensus clustering with the K-means method measured by the Euclidean metric demonstrated k = 3 was an optional clustering number: 125 samples in Cluster A (low immunity), 203 samples in Cluster B (median immunity), and 185 samples in Cluster C (high immunity) (Figure 2C, Supplementary Table S3). Survival analysis indicated that patients in Cluster A had the poorest prognosis while those in Cluster B had relatively favorable outcomes (*p* < 0.001, Figure 2D). The chi-squared test indicated that survival status (*p* < 0.001), gender (*p* < 0.001), pathologic stage (*p* < 0.05), and tumor status (*p* < 0.01) had significant differences among the clustering subtypes (Figure 2E). As shown in Figures 2F–H, immune score, stromal score, and ESTIMATE score increased gradually and significantly from Cluster A to Cluster C (*p* < 0.001).

### 3.2 Biological pathway enrichment and tumor immune infiltration among clusters

**FIGURE 3:**
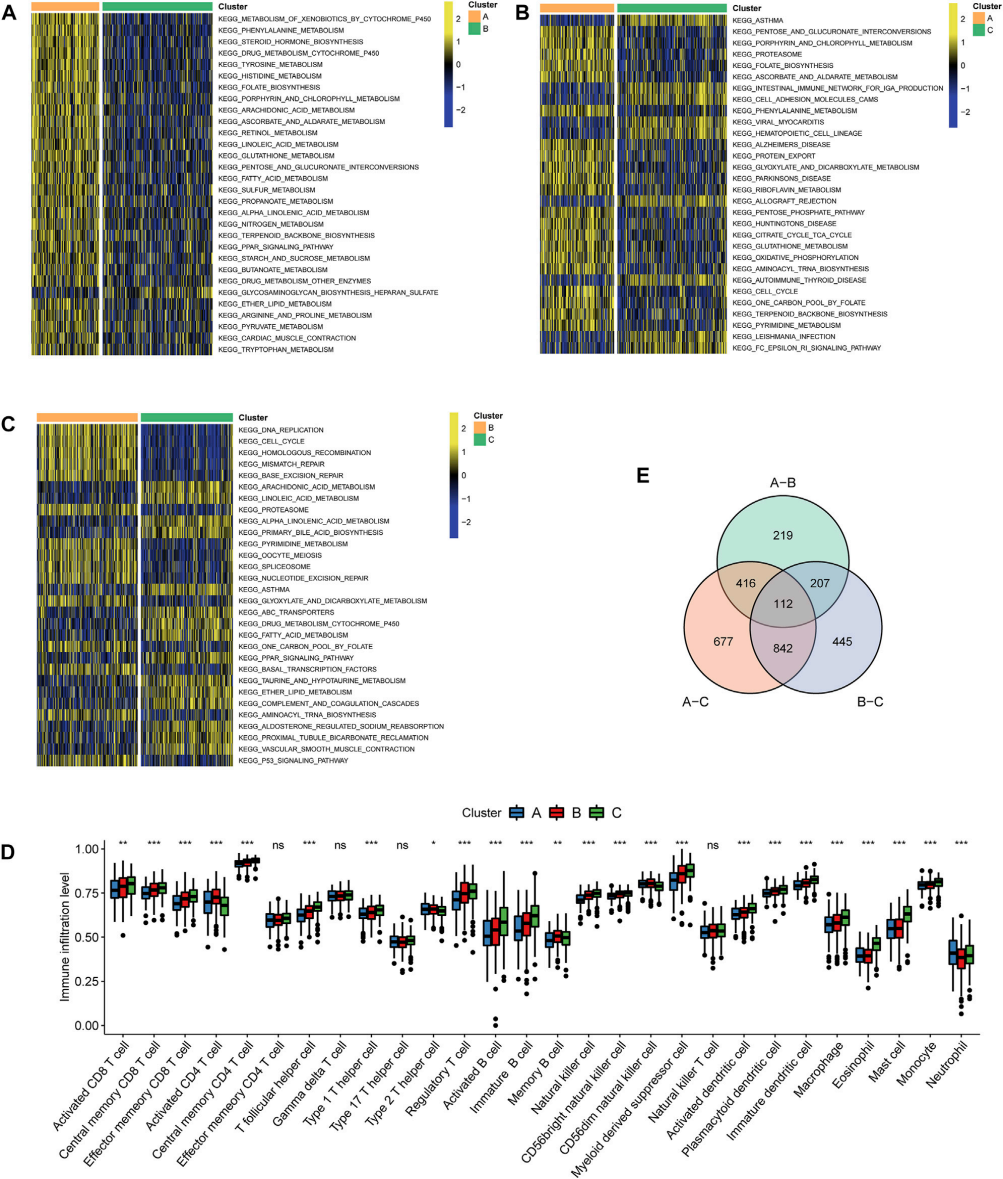
Characteristics of biological pathways and immune-infiltrating levels among clustering subtypes. Gene set variation analysis for Kyoto Encyclopedia of Genes and Genomes pathways between **(A)** Cluster A and Cluster B, **(B)** Cluster A and Cluster C, and **(C)** Cluster B and Cluster **(C)**. Yellow color represented active pathways, while blue color represented inhibited pathways. **(D)** Overlapped differentially expressed genes between each of the two clustering subtypes are shown by a Venn diagram. **(E)** Tumor immune-infiltrating levels of 28 cell types predicted by single-sample gene set enrichment analysis (***p < 0.001, **p < 0.01, and *p < 0.05; ns: no significance).

GSVA functional enrichment revealed that Cluster A was markedly enriched in pathways such as xenobiotic metabolism mediated by cytochrome P450, phenylalanine metabolism, steroid hormone biosynthesis, and drug metabolism induced by cytochrome P450 compared with Cluster B (Figure 3A); pathways including pentose and glucoronate interconversions, porphyrin metabolism, and proteasome were active in Cluster A, while several pathways of asthma, intestinal immune networks for immunoglobulin A production, and cell adhesion molecule cams were active in Cluster C (Figure 3B). DNA replication, cell cycle, and homologous recombination pathways were active in Cluster B, while Cluster C presented enrichment pathways of arachidonic acid metabolism, linoleic acid metabolism, and primary bile acid biosynthesis (Figure 3C). The results of REACTOME pathway enrichment are provided in Supplementary Figures S1A–C. Variations in tumor immune-infiltrating levels of 28 cell types were estimated by the KW test. It was demonstrated that Cluster C had significantly higher infiltrating levels of the most cell types, such as CD8+cells, regulatory T cell, activated B cell, and natural killer cells (Figure 3D). The differentially expressed strategy determined 112 DEGs between each of the two clusters was also acquired (Figure 3E, Supplementary Figure S2A; Supplementary Table S4).

### 3.3 Feature selection and prognostic risk model development

**FIGURE 4:**
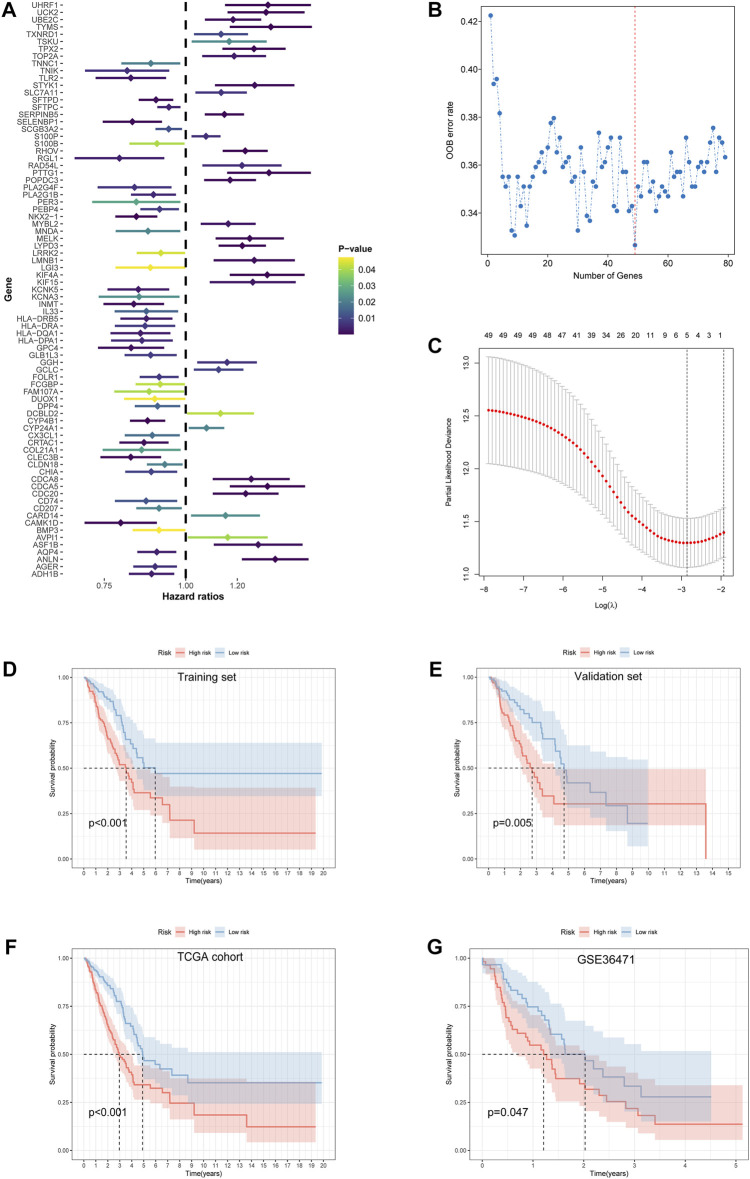
Feature selection and development of the prognostic risk model. **(A)** Univariate Cox proportional hazard regression with 112 differentially expressed ramRNAs. **(B)** The relationship of the “out-of-bag” error rate and gene numbers included in the random forest model by the method of sliding window sequential forward feature selection. **(C)** Solution paths of partial likelihood deviance and lambda in the least absolute shrinkage and selection operator Cox regression model. Left black dotted lines represented the minimum lambda and its corresponding number of genes, which was optimal. **(D–G)** Survival differences between the low- and high-risk groups in the training set, validation set, TCGA cohort, and GSE36471 estimated by the log-rank test. TCGA: The Cancer Genome Atlas.

A univariate Cox proportional hazard regression screened for 79 signatures with *p* < 0.05 out of 112 DEGs (Figure 4A). Dimension reduction was further carried out by weighted random forests and sliding window sequential forward feature selection. It was demonstrated that the OOB error reached its minimum with 49 genes (Figure 4B). The relative importance of variables is illustrated in Supplementary Figure S2B. The entire TCGA cohort was randomly divided into a training set and a validation set. Pearson’s chi-squared test was adopted to examine the clinicopathological difference between the two inner separate sets (Table 1). Based on the training set, LASSO Cox regression determined five optimal signatures (ANLN, HLA-DQA1, RHOV, TLR2, and TYMS) at the minimum partial likelihood deviance for LUAD prognosis (Figure 4C). Then, patients from both TCGA cohort and GEO cohort were classified into low- and high-risk groups at the cutoff of 2.198, which was the median value of the risk score in the training set.

**TABLE 1:** Pearson’s chi-squared test with clinicopathological information for three LUAD sets in the TCGA cohort.

Covariate	Type	Entire	Test	Training	*p* value
Vital status	Alive	312 (63.67%)	119 (61.34%)	193 (65.2%)	0.385
	Dead	178 (36.33%)	75 (38.66%)	103 (34.8%)	
Age	≤60	153 (31.22%)	56 (28.87%)	97 (32.77%)	0.658
	>60	327 (66.73%)	134 (69.07%)	193 (65.2%)	
	Unknown	10 (2.04%)	4 (2.06%)	6 (2.03%)	
Gender	Female	262 (53.47%)	102 (52.58%)	160 (54.05%)	0.749
	Male	228 (46.53%)	92 (47.42%)	136 (45.95%)	
Stage	Stage I–II	378 (77.14%)	157 (80.93%)	221 (74.66%)	0.127
	Stage III–IV	104 (21.22%)	36 (18.56%)	68 (22.97%)	
	Unknown	8 (1.63%)	1 (0.52%)	7 (2.36%)	
T	T1–T2	426 (86.94%)	172 (88.66%)	254 (85.81%)	0.331
	T3–T4	61 (12.45%)	20 (10.31%)	41 (13.85%)	
	Unknown	3 (0.61%)	2 (1.03%)	1 (0.34%)	
N	N0–N1	409 (83.47%)	163 (84.02%)	246 (83.11%)	0.174
	N2–N3	70 (14.29%)	24 (12.37%)	46 (15.54%)	
	Unknown	11 (2.24%)	7 (3.61%)	4 (1.35%)	
Tumor status	Tumor free	275 (56.12%)	109 (56.19%)	166 (56.08%)	0.461
	Unknown	50 (10.2%)	16 (8.25%)	34 (11.49%)	
	With tumor	165 (33.67%)	69 (35.57%)	96 (32.43%)	

Numbers in the bracket represented the ratio of the corresponding sample numbers.

### 3.4 Validation of model effectiveness and construction of a nomogram model

**FIGURE 5:**
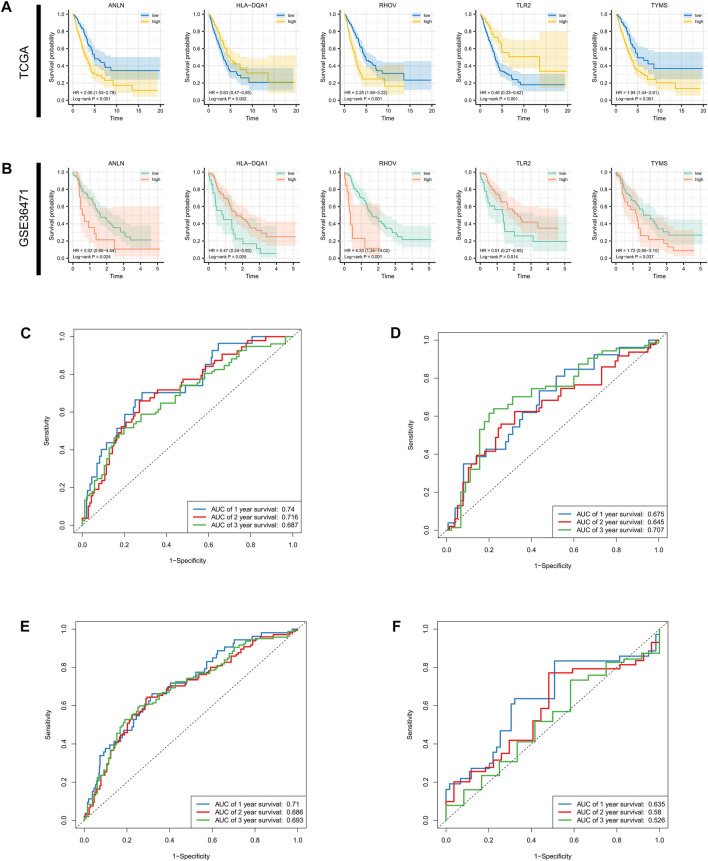
Survival analysis for single gene and AUC estimation. Survival differences evaluated by log-rank test between the low- and high-expression groups at the optimal cutoff of each optimal prognostic signature in the **(A)** entire TCGA cohort and **(B)** GSE36471. **(C–F)** 1-, 3-, and 5-year receiver operating characteristic curves and AUCs in the training set validation set, TCGA cohort, and GSE36471. AUC: area under the curve; TCGA: The Cancer Genome Atlas.

**FIGURE 6:**
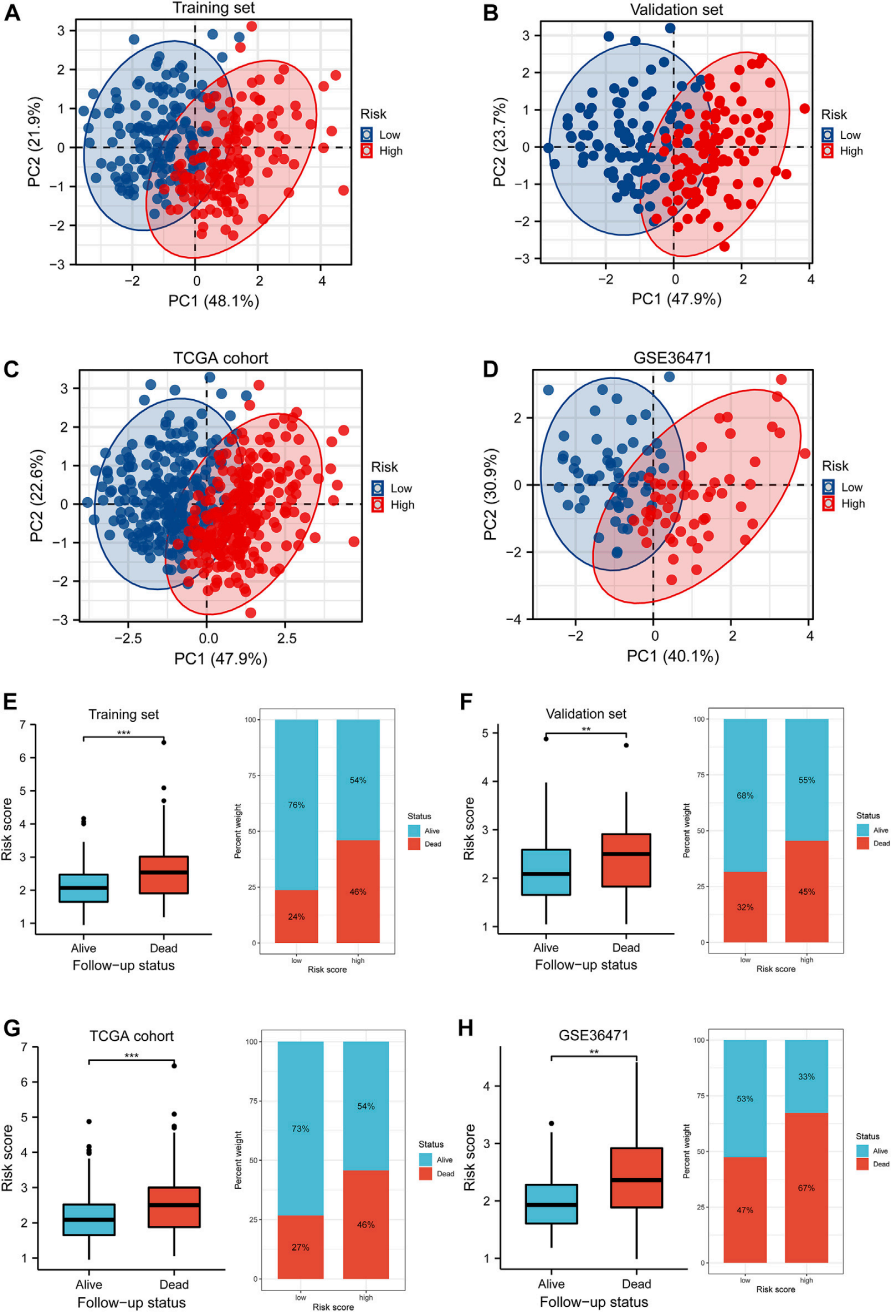
PCA and differences of the survival status with the risk score. **(A–D)** Distributions of low- and high-risk patients in the PCA for the training set, validation set, TCGA cohort, and GSE36471 dataset. The number in the brackets represented the ratio of explaining variability of the whole data. **(E–H)** The Wilcoxon rank-sum test implied the variations of risk scores between the alive and dead follow-up status in the training set, validation set, TCGA cohort, and GSE36471. PCA: principle component analysis.

Survival differences between the low- and high-risk groups in the training set (*p* < 0.001, Figure 4D), validation set (*p* = 0.005, Figure 4E), entire TCGA cohort (*p* < 0.001, Figure 4F), and GSE36471 (*p* = 0.047, Figure 4G) revealed that patients in the high-risk group experienced poor prognosis compared with the low-risk group. Simultaneously, the log-rank test of low- and high-expression groups with each optimal signature suggested that overexpressed ANLN, RHOV, and TYMS led to worse survival, while upregulation of HLA-DQA1 and TLR2 led to favorable prognosis (Figures 5A,B). ROC curves and AUC values of 1-, 2-, and 3-year survival in the training set (Figure 5C), validation set (Figure 5D), entire TCGA cohort (Figure 5E), and GSE36471 (Figure 5F) are shown. PCA demonstrated that the cumulative value of principal component 1 (PC1) and principal component 2 (PC2), which was over 70%, sufficiently explained the variance of the signature expression levels (Figures 6A–D). Hence, it was notable that patients in the different risk groups were stratified effectively. Patients in the high-risk group had higher mortality rates (training set: *p* < 0.001; validation set: *p* < 0.01; TCGA cohort: *p* < 0.001; GSE36471: *p* < 0.01, Figures 6E–H), and ANLN, RHOV, and TYMS overexpressed in the high-risk group, while the expression of HLA-DQA1 and TLR2 were reduced in the high-risk group (Supplementary Figures 3A–D). Independent prognostic analysis demonstrated that the tumor status and risk score could serve as an independent factor for predicting LUAD patients’ prognosis in the training set, validation set, and entire TCGA cohort (Table 2). Also, KM survival analysis of different clinicopathological variables showed that patients of the high-risk group had significantly poor prognosis in all subgroups including age, gender, pathological stage, and tumor status (Supplementary Figure S4). It should be noted that the survival of patients with advanced stages showed no statistical significance (Supplementary Figures 4C–E). We also found that male or advanced patients exhibited significantly higher risk scores than the other populations (Supplementary Figures 5A–D).

**TABLE 2:** Independent prognostic analysis for clinicopathological variables and risk scores by Cox regression.

Dataset	Factor	Univariate			Multivariate		
		*p* value	HR	95%CI	*p* value	HR	95%CI
Training	Age	0.501	0.993	0.972–1.014			
	Gender	0.982	1.005	0.636–1.588			
	Stage	<0.001	1.674	1.343–2.087	0.833	1.035	0.753–1.421
	T	0.006	1.466	1.116–1.925	0.240	1.208	0.882–1.655
	N	0.001	1.536	1.192–1.981	0.088	1.359	0.956–1.932
	Tumor status	<0.001	7.085	4.147–12.105	<0.001	6.164	3.502–10.849
	Risk score	<0.001	2.032	1.594–2.589	<0.001	1.608	1.266–2.042
Validation	Age	0.393	1.012	0.985–1.039			
	Gender	0.526	1.179	0.708–1.962			
	Stage	0.001	1.534	1.192–1.975	0.913	0.977	0.642–1.486
	T	0.001	2.013	1.344–3.013	0.118	1.466	0.907–2.371
	N	0.001	1.667	1.243–2.236	0.054	1.591	0.992–2.551
	Tumor status	<0.001	5.594	3.027–10.337	<0.001	5.813	3.063–11.029
	Risk score	0.002	1.637	1.201–2.232	0.014	1.619	1.101–2.382
TCGA	Age	0.914	1.001	0.984–1.018			
	Gender	0.607	1.093	0.780–1.531			
	Stage	<0.001	1.576	1.340–1.854	0.894	0.983	0.769–1.258
	T	<0.001	1.572	1.265–1.954	0.079	1.251	0.974–1.606
	N	<0.001	1.566	1.294–1.896	0.008	1.442	1.101–1.888
	Tumor status	<0.001	6.465	4.325–9.664	<0.001	6.246	4.119–9.471
	Risk score	<0.001	1.885	1.557–2.283	<0.001	1.633	1.350–1.975

**FIGURE 7:**
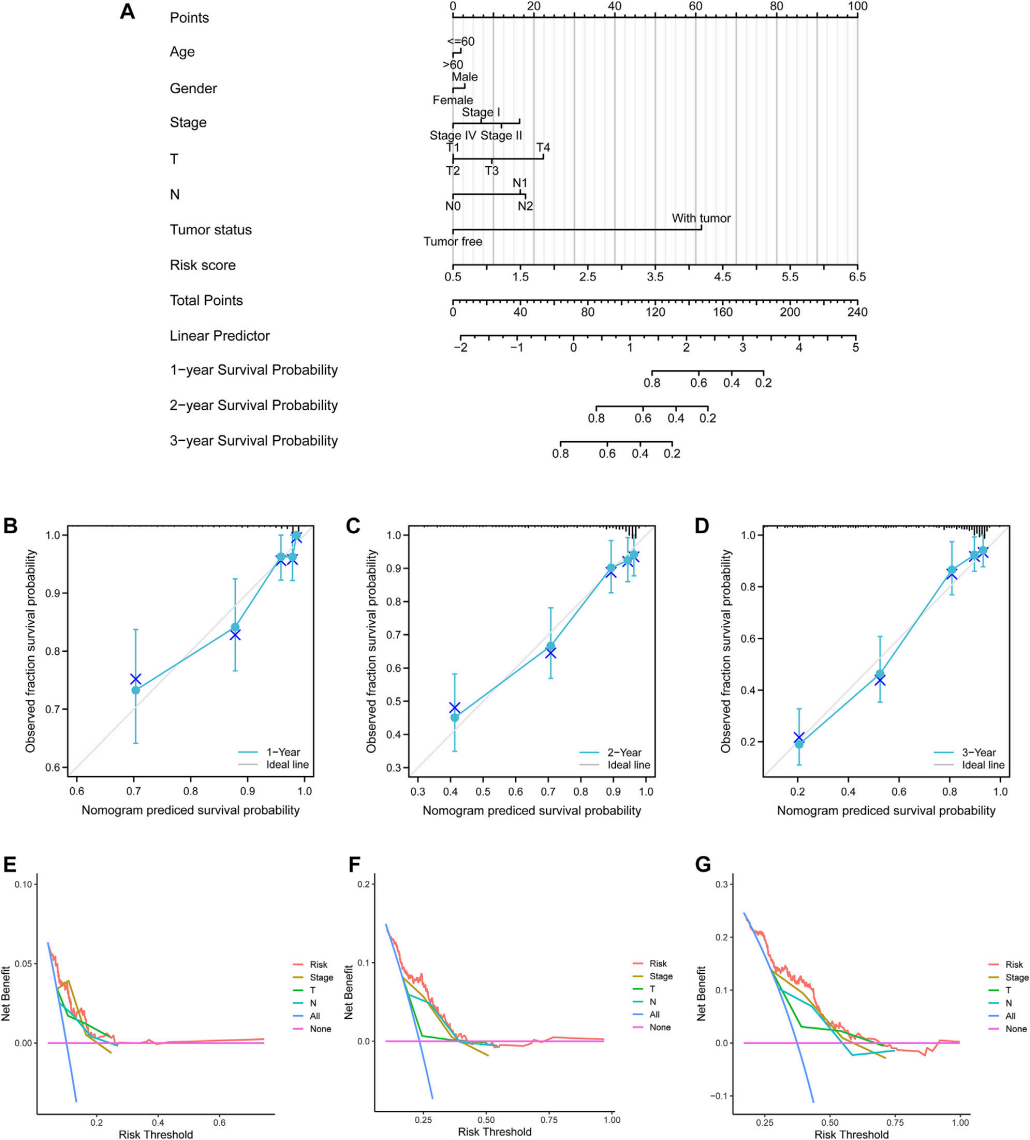
Nomogram model and decision curve analysis curves. **(A)** A nomogram model to predict lung adenocarcinoma patients’ survival with clinicopathological variables and risk scores. **(B–D)** 1-, 2-, and 3-year calibration curves. Blue crosses represented the result of each point after the stratified Kaplan–Meier correction. Small gray vertical lines at the top of the boxes represented the distribution of survival rates under the model predictions. **(E–G)** 1-, 2-, and 3-year survival probability decision curve analysis curves for the pathological stage, T stage, N stage, and risk score.

A nomogram model with age, gender, pathological stage, T stage, N stage, tumor status, and risk score were established (Figure 7A). The predictive accuracy of the nomogram was determined by 1-, 2-, and 3-year survival probability calibration curves (Figures 7B–D) and C-index of 0.786 (95% confidence interval (CI) = 0.765–0.808), showing favorable fitness and discrimination ability. In addition, compared with the conventional TNM stage, the risk score exhibited higher net benefits of 1-, 2-, and 3- year survival probability (Figures 7E–G), which indicated that risk scores might have optimal prognostic values.

### 3.5 Profiles of cluster and mutation risks

**FIGURE 8:**
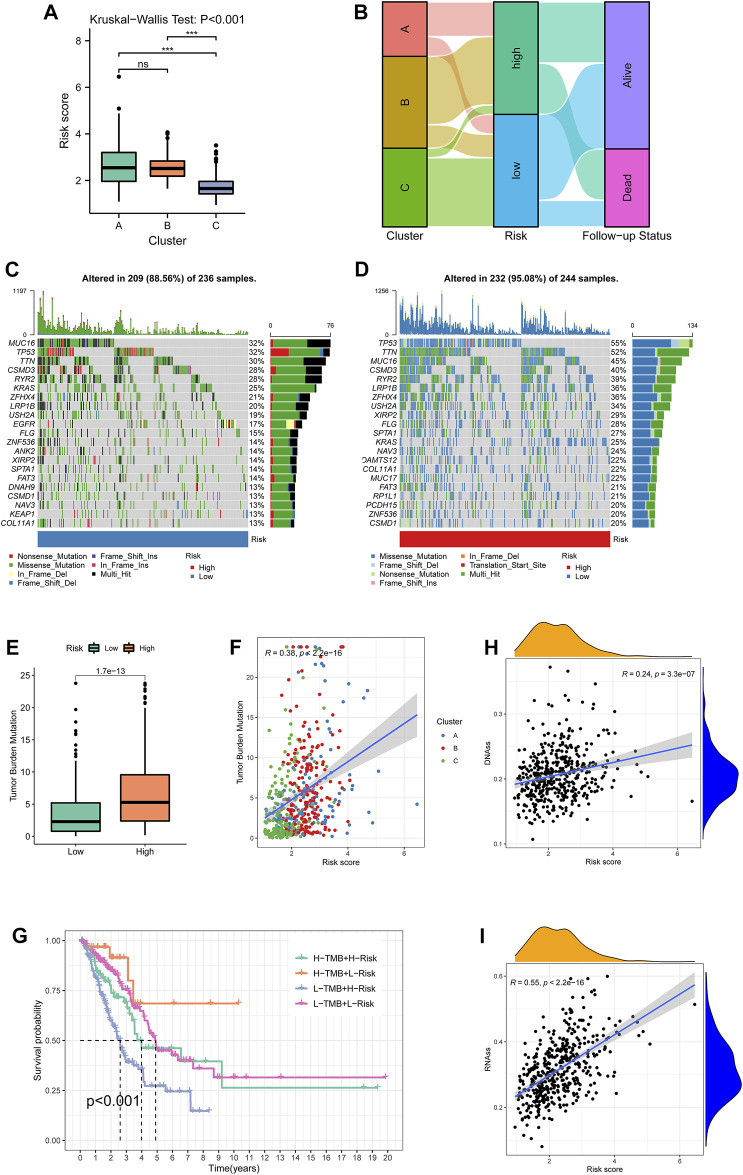
Cluster-risk and mutation-risk profiles. **(A)** Variations among the three clustering subtypes revealed by the Kruskal–Wallis test. **(B)** An alluvial diagram illustrated the distributions among clusters, risk groups, and vital status. **(C–D)** The top 20 mutated genes in the low- and high-risk groups. The upper bar plot showed the total mutation frequency of each sample. The number on the right indicated the mutation frequency in each gene. The right bar plot showed the proportion of each variant type. The stacked stripe below showed risk subtypes, blue for low risk and red for high risk. **(E)** TMB differences between the low- and high-risk groups. **(F)** The correlation of TMB and risk scores. Points with three colors represented different clustering subtypes. **(G)** Survival differences among four TMB-risk stratifications. **(H, I)**Correlation between the risk score and tumor purity estimated by RNA-seq and DNA methylation. TMB: tumor mutational burden.

The KW test demonstrated that Cluster C exhibited the lowest risk score (*p* < 0.001, Figure 8A). The distribution of different clusters among risk subtypes and the follow-up status are shown by an alluvial diagram in Figure 8B. By summarizing the mutation information including frequency and types, the mutation landscape in the low- and high-risk groups was manifested, respectively, by an oncoplot (Figures 8C,D). It was notable that MUC16, TP53, and TNN exhibited the highest frequency in the low-risk group. MUC16 and TP53 showed mostly non-sense mutation, while TP53 had the most missense mutation. Then, TMB of each sample was calculated, and Figure 8E evinced that patients in the high-risk group had higher TMB compared with the low-risk group (*p* < 0.001). TMB was also positively correlated with the risk score with the coefficient of 0.38 and *p* < 0.001 (Figure 8F). After separating samples into four novel TMB-risk subtypes, their survival differences were analyzed. It was noticeable that patients with high-TMB and low-risk showed better prognosis while patients with low-TMB and high-risk experienced the poorest survival outcomes (Figure 8G). Furthermore, stemness indices measured by DNAss (Figure 8H) and RNAss (Figure 8I) also had a significantly positive connection with the risk score (*p* < 0.001).

### 3.6 Associations of the TME, immunotherapies, and risk model

**FIGURE 9:**
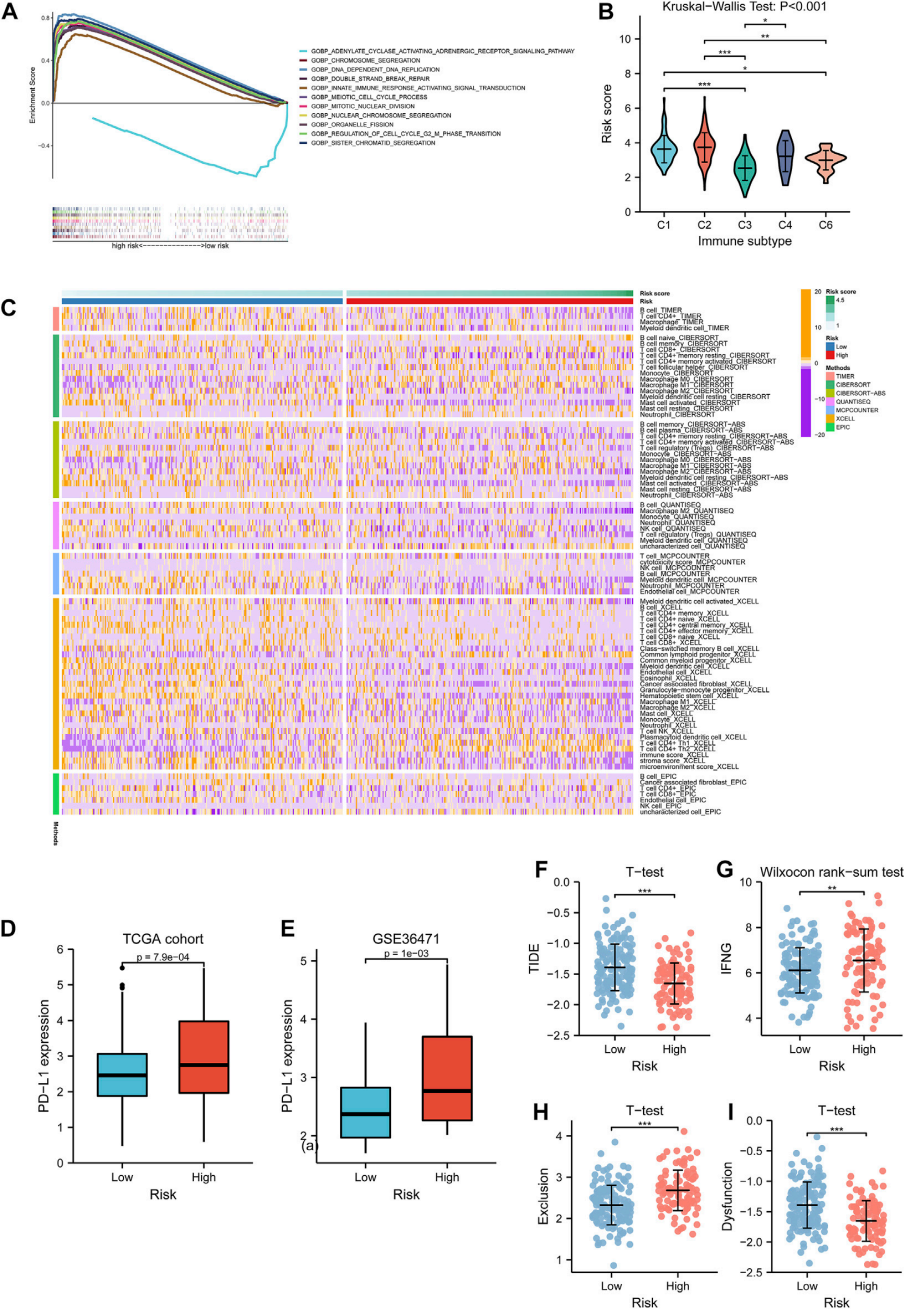
Tumor immune microenvironment investigation. **(A)** Biological functional analysis of biological processes for the risk subtypes. The curves upon the horizontal axis represented terms enriching in the high-risk group, while the curve beneath the horizontal axis represented terms enriching in the low-risk group. Stripes below showed the gene abundance of each function annotation. **(B)** Differences of risk score among five immune subtypes in lung adenocarcinoma. **(C)** Associations of immune-infiltrating levels of distinctive cell types calculated by several methods and risk scores are shown by a heatmap. **(D, E)** PD-L1 expression variations between the low- and high-risk group in TCGA cohort and GSE36471. **(F–I)** Relationships between TIDE score, IFNG score, T-cell exclusion score, T-cell dysfunction score, and risk subtypes respectively. TCGA: The Cancer Genome Atlas.

GSEA functional enrichment for biological processes indicated that the term “adenylate cyclase activating adrenergic receptor signaling pathway” was active in the low-risk group, while high risk manifested significant terms such as chromosome segregation, DNA dependent DNA replication, organelle fission, and innate immune response activating signal transduction (Figure 9A). With the information regarding immunophenotypes of TCGA cohort retrieved from UCSC Xena, it could be observed that C2 held the highest risk score, and the five immune subtypes had significantly different score levels (*p* < 0.001, Figure 9B). The evidence from the TIMER database demonstrated that the immune-infiltration abundance of cell types such as macrophage M0/1 (by CIBERSORT) and T cell CD4^+^ Th1/2 (by XCELL) was positively associated with the risk score while that of B cells (by TIMER), activated mast cells (by CIBERSORT), and M2 macrophages (by QUANTISEQ) were negatively associated with the risk score (Figure 9C). To explore anti-PD-L1 therapeutic effectiveness, PD-L1 expression was analyzed between the low- and high-risk groups. The Wilcoxon rank-sum test implied that PD-L1 was overexpressed in the high-risk group than the low-risk group from both TCGA cohort (*p* < 0.001, Figure 9D) and GEO cohort (*p* = 0.001, Figure 9E). In addition, immunotherapy examination according to the TIDE prediction score represented the possibility that patients with high-risk enjoying significantly lower TIDE scores (*p* < 0.001, Figure 9F) were insusceptible to immune evasion, which suggested that the patients were more likely to benefit from therapy with ICIs. Furthermore, the IFNG score (*p* < 0.01, Figure 9G) and T-cell exclusion score showed higher level in the high-risk group (*p* < 0.001, Figure 9H), while T-cell dysfunction score exhibited lower level in the high-risk group (*p* < 0.001, Figure 9I).

### 3.7 Probes into drug sensitivity, risk, and optimal signatures

**FIGURE 10:**
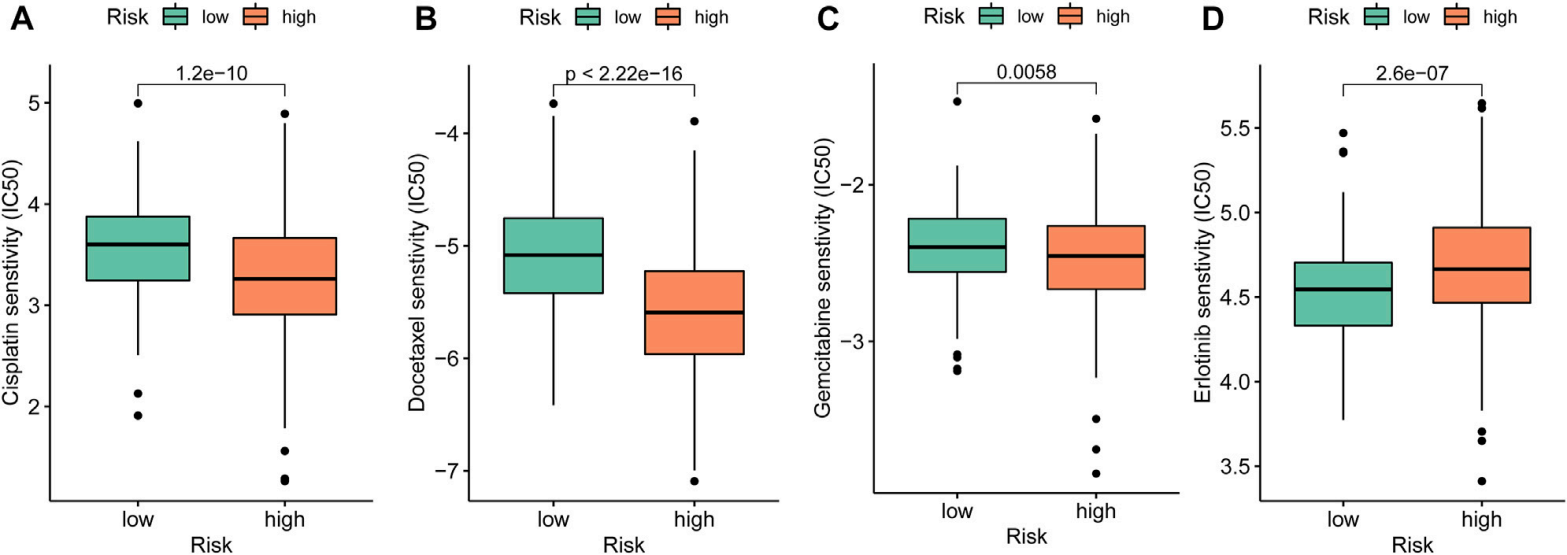
Drug sensitivity exploration. **(A–D)** Chemosensitivity measured by IC_50_ of cisplatin, docetaxel, gemcitabine, and erlotinib for anti-tumor therapies.

Chemosensitivity of drugs for anti-LUAD therapies such as cisplatin (*p* < 0.001), docetaxel (*p* < 0.001), and gemcitabine (*p* = 0.006) had significantly lower IC_50_ in the high-risk group (Figures 10A–C), while erlotinib (*p* < 0.001) held higher IC_50_ in the high-risk group (Figure 10D).

## 4 Discussion

In this study, differentially expressed survival-related ramRNAs were screened to identify the novel subtypes of LUAD with consensus clustering based on the TCGA dataset. Patients were classified into three groups, and there were statistical differences in KM survival analysis. GSVA functional enrichment analysis for distinct subtypes revealed that metabolic pathways such as phenylalanine, tyrosine, and histidine were activated in Cluster A, which promoted essential amino acid metabolism and tumorigenesis (Yue et al., 2017); cytogenetic pathways including DNA replication, cell cycle, homologous recombination, and mismatch repairs were enriched in Cluster B, which were tightly associated with tumorigenesis (Zong et al., 2016; McGrail et al., 2020). Also, immune-related pathways contain intestinal immune networks for IgA production, cell adhesion molecule cams, and FC epsilon RI signaling activated in Cluster C, which were related to the response of inflammatory cells (Palm et al., 2014; Inman et al., 2018). To investigate the differences of immune-infiltrating levels among the subtypes, the ssGSEA algorithm was adopted and it found that Cluster C exhibited higher infiltrating abundance in most cell types.

DEGs were further determined between each of the two clusters and obtained overlapped targets for feature selection. Subsequently, by integrating both conventional statistical methods like univariate Cox regression and machine learning strategy including weighted random forest with sliding window sequential forward feature selection, we reduced the dimension of the data structure for LASSO regression to develop an effective prognostic risk model with the median cutoff. It was illustrated that high-risk patients had poorer survival outcomes compared with low-risk patients, which was consistent in the training set, validation set, and GEO cohort. Also, each optimal signature showed differences in KM survival for LUAD patients. Thus, these agents can be potential biomarkers for prognostic prediction. ROC analysis demonstrated that AUCs reached 0.70, indicating that our model had promising accuracy and sensitivity. PCA also demonstrated an optimal discrimination of risk.

Nomograms have been acknowledged as a tailored tool with a single numerical prediction of the probability of an event for cancer prognosis (Iasonos et al., 2008). This study constructed a nomogram with age, gender, pathologic stage, T stage, N stage, tumor status, and risk scores to estimate the 1-, 2-, or 3-year survival probability of LUAD patients. The C-index of 0.786 implied that the evaluation of the model had a strong consistency with actual outcomes. The risk score also underwent higher net benefit than the conventional stage system according to DCA, which suggested risk had values of prognostic prediction. In addition, it was showed that male patients had higher risk scores than female ones, which are consistent with previous reports (Hsu et al., 2015; Hsu et al., 2017). Therefore, male donors exhibited lower immune-infiltrating levels (Figure 8A), which suggested potentially insensitive responses to immunotherapy. Although advanced patients were characterized by high risk, the survival distinction did not show significance. We reasoned that the predisposition of survival differences among advanced individuals includes not only the redox hallmark quantified by the transcriptome in our study, but also other genetic and microenvironment cofounders, such as immunological and metabolic factors.

Then, the correlations were explored among clustering subtypes, risk, and mutation characteristics. The KW test showed patients from Cluster C had the lowest risk score and better survival, which fit well with the KM result of clustering subtypes. Previous studies demonstrated that MUC16 mutation associated with response to immune checkpoint inhibitors in solid tumors and TP53 mutation status had potential predictive values for response to PD-1 blockade immunotherapy in LUAD (Dong et al., 2017; Zhang et al., 2020). Our results revealed that high-risk groups had higher total mutation frequency as well as TP53, TTN, and MUC16, which demonstrated that patients with higher risk score may benefit from ICI treatment. Moreover, it was found that TMB was positively correlated with the risk score, which indicated that high-risk patients are more likely benefited from anti-PD-1 therapies and is consist with the previous study (Marabelle et al., 2020). However, several studies found that TMB was not markedly associated with the PD-1/PD-L1 expression (Hellmann et al., 2018; Rizvi et al., 2018). Hence, to further disclose the important value of the risk score in immune checkpoint therapies, the relationship between the TME and risk score was analyzed.

Immune regulation is pivotal to the development and progression of LUAD affected by the proportion of infiltrating immune cells (Yao et al., 2021). The risk score varied significantly within the six immune subtypes. Accordingly, this study then downloaded the information of immune-infiltrating of various cell types measured by different methods and detected the correlations of risk and tumor immune fractions. It could be observed that the risk score was positively connected with the immune-infiltrating levels of cells such as M0/M1 macrophages , plasmacytoid dentritic cells, and CD4^+^ Th1/Th2, whereas the risk score was negatively correlated with cells such as B cells, neutrophils, NK cells, and T-cell regulatory. These reflected that high-risk LUAD patients had stronger immunosuppression and weaker immunoreactivity.

TIDE is an inventive computational protocol to screen two factors, including the induction of T-cell dysfunction and the prevention of T-cell infiltration in tumor tissues, which has been validated as an effective biomarker of prognostic prediction (Jiang et al., 2018). Our research study found that patients in the high-risk group had the lower TIDE score and patients in the low-risk group had the lower T-cell exclusion score but the higher T-cell dysfunction score. It suggested that patients were more unlikely to experience immune escape, which had more opportunities to benefit from ICI therapies, and the insensitive ICI response may result from immune evasion T-cell dysfunction rather than T-cell exclusion. ICI therapies targeting several immunological checkpoint blockades have achieved prospective clinical benefits in treating LUAD patients (Chen et al., 2021). In the current study, PD-L1 was significantly overexpressed in the high-risk group of both TCGA dataset and the independent cohort GSE36471 dataset. Therefore, patients with higher risk scores could be more sensitive to PD-1/PD-L1 blockades and thus the individuals may benefit from ICI treatment (Ansell et al., 2015; Galluzzi et al., 2018).

Finally, drug sensitivity analyses implied that the low-risk group was related to higher IC_50_ of chemotherapeutics such as cisplatin, docetaxel, and gemcitabine, while erlotinib had higher IC_50_ in the high-risk group. The aforementioned results indicated these signatures of out model might play a vital role in tumor cell sensitivity or resistance. However, as the database is limited, the half maximal inhibitory concentration of more target therapy and immunotherapy drugs are not investigated in this study and requires more research to confirm and explore the findings.

However, some limitations should be considered in this study. First, we only used retrospective datasets for this study, and a multi-center prospective clinical cohort of LUAD samples is a necessity to verify the stability of phenotyping. In addition, further experiments are still needed to investigate the relationship between the redox signature and immunotherapy and reveal how ramRNAs affect genomic instability. In the future, the combination of ramRNAs and immunotherapy may be a promising strategy for LUAD treatment.

## 5 Conclusion

In conclusion, this study successfully constructed a novel predictive signature of lung adenocarcinoma based on redox-associated genes, which had optimal prognostic values and affected TME cell-infiltrating characterization. Moreover, the model helps researchers understand the association between redox and tumorigenesis. This study contributes to guiding more effective strategies for TMB and anti-tumor immunotherapy.

## Data Availability

The original contributions presented in the study are included in the article/Supplementary Material; further inquiries can be directed to the corresponding authors.

## Abbreviations

AUC, area under the curve; C-index, concordance index; CI, confidence interval; DCA, decision curve analysis; DEG, differentially expressed gene; DNAss, DNA stemness score; ESTIMATE, Estimation of Stromal and Immune cells in Malignant Tumors using Expression data; FC, fold change; GEO, Gene Expression Omnibus; GSVA, gene set variation analysis; ICI, immune checkpoint inhibitor; KM, Kaplan–Meier; KW, Kruskal–Wallis; LASSO, least absolute shrinkage and selection operator; LUAD, lung adenocarcinoma; MSigDB, Molecular Signatures Database; NSCLC, non-small-cell lung cancer; OOB, out-of-bag; ramRNA, redox-associated messenger RNA; RNAss, RNA stemness score; ROC, receiver operating characteristic; ROS, reactive oxygen species; SNV, single-nucleotide variants; ssGSEA, single-sample Gene Set Enrichment Analysis; TCGA, The Cancer Genome Atlas; TMB, tumor mutational burden; TME, tumor microenvironment.
