# Supplementary figures and images for: Redox-associated messenger RNAs identify novel prognostic values and influence the tumor immune microenvironment of lung adenocarcinoma
Source: Front Genet. 2023 Feb 16;14:1079035. doi: 10.3389/fgene.2023.1079035 (PMC9977811; doi:10.3389/fgene.2023.1079035)

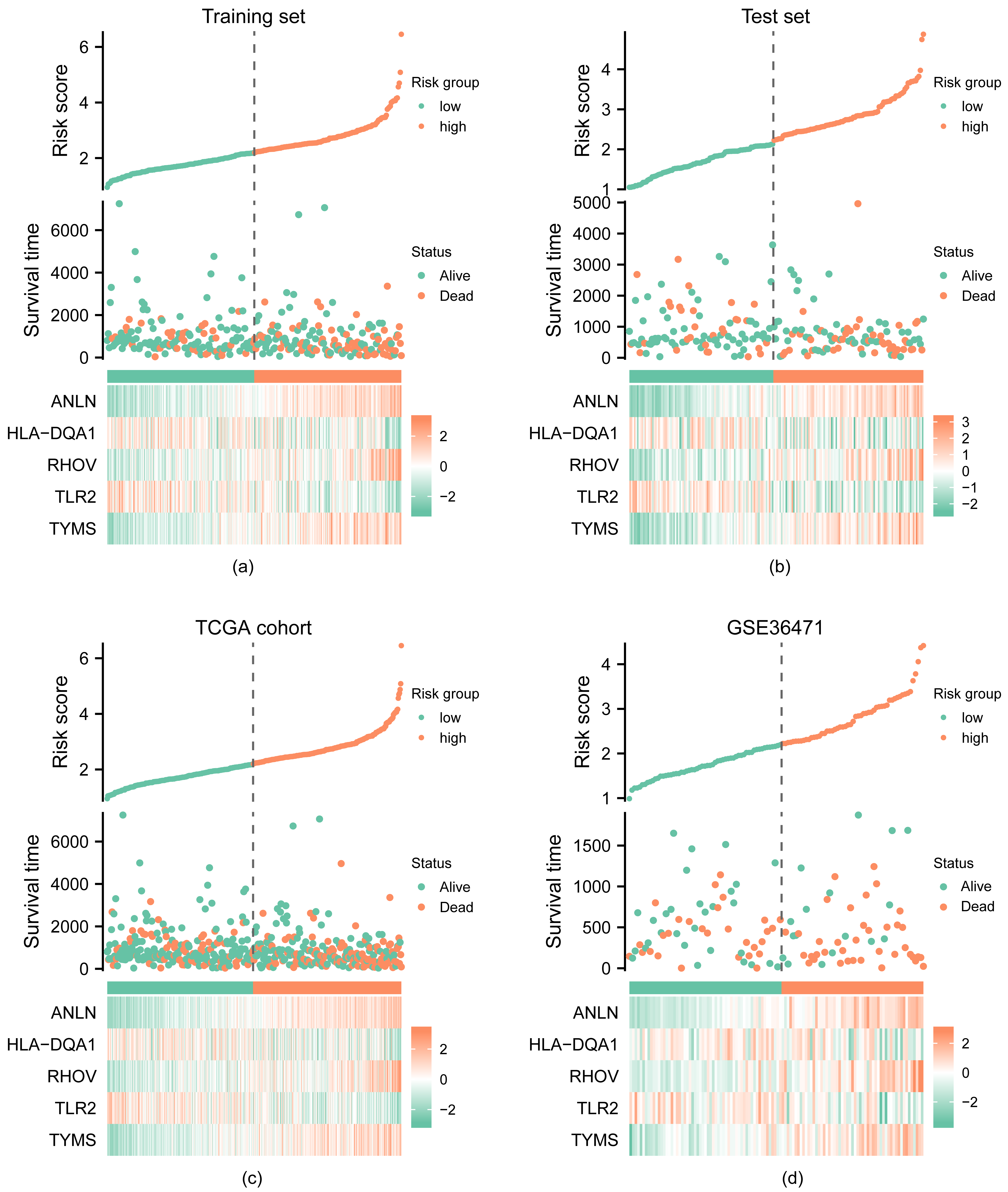

Supplement: Supplementary file 3 [file Image3.tif]

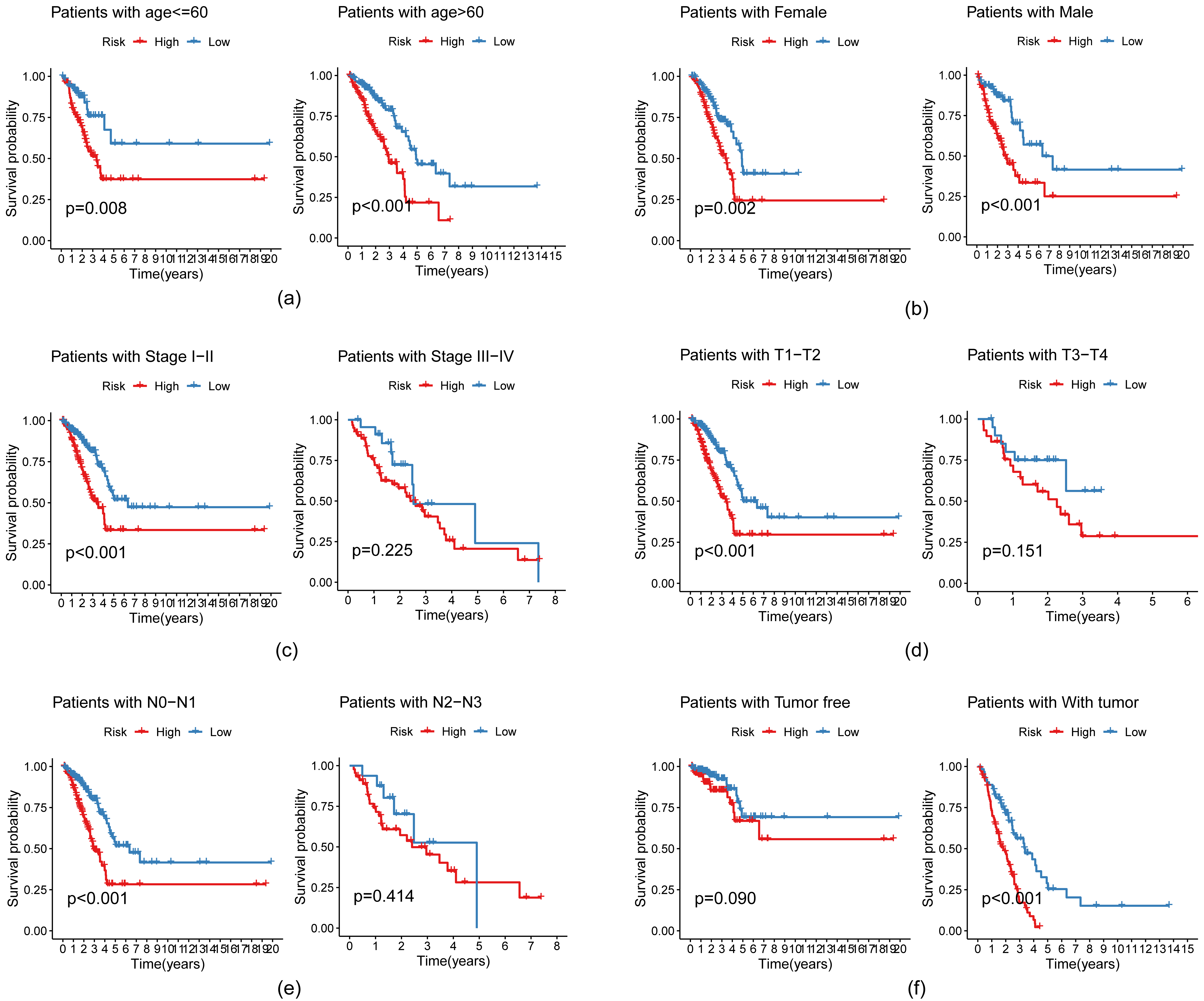

Supplement: Supplementary file 4 [file Image4.tif]

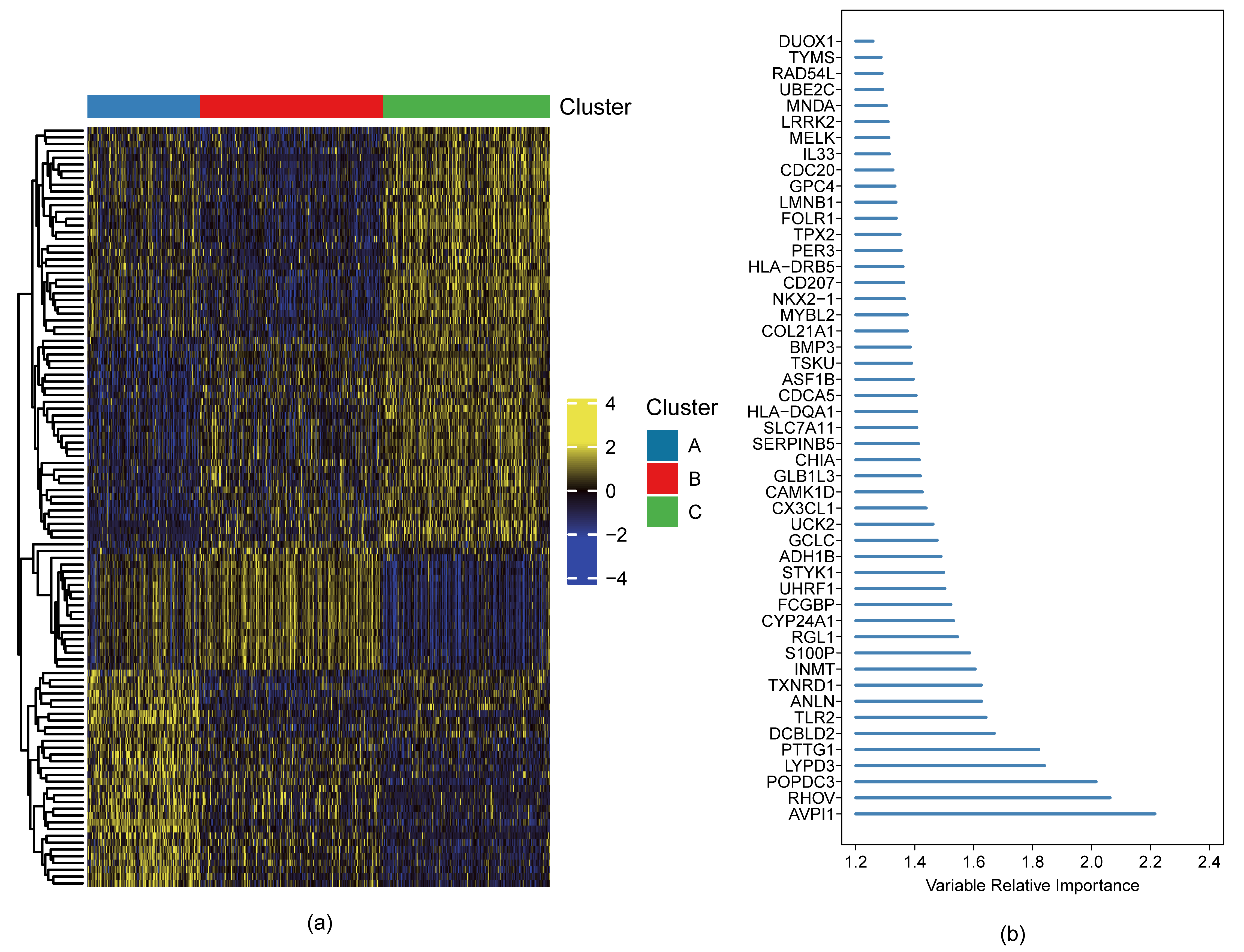

Supplement: Supplementary file 5 [file Image2.tif]

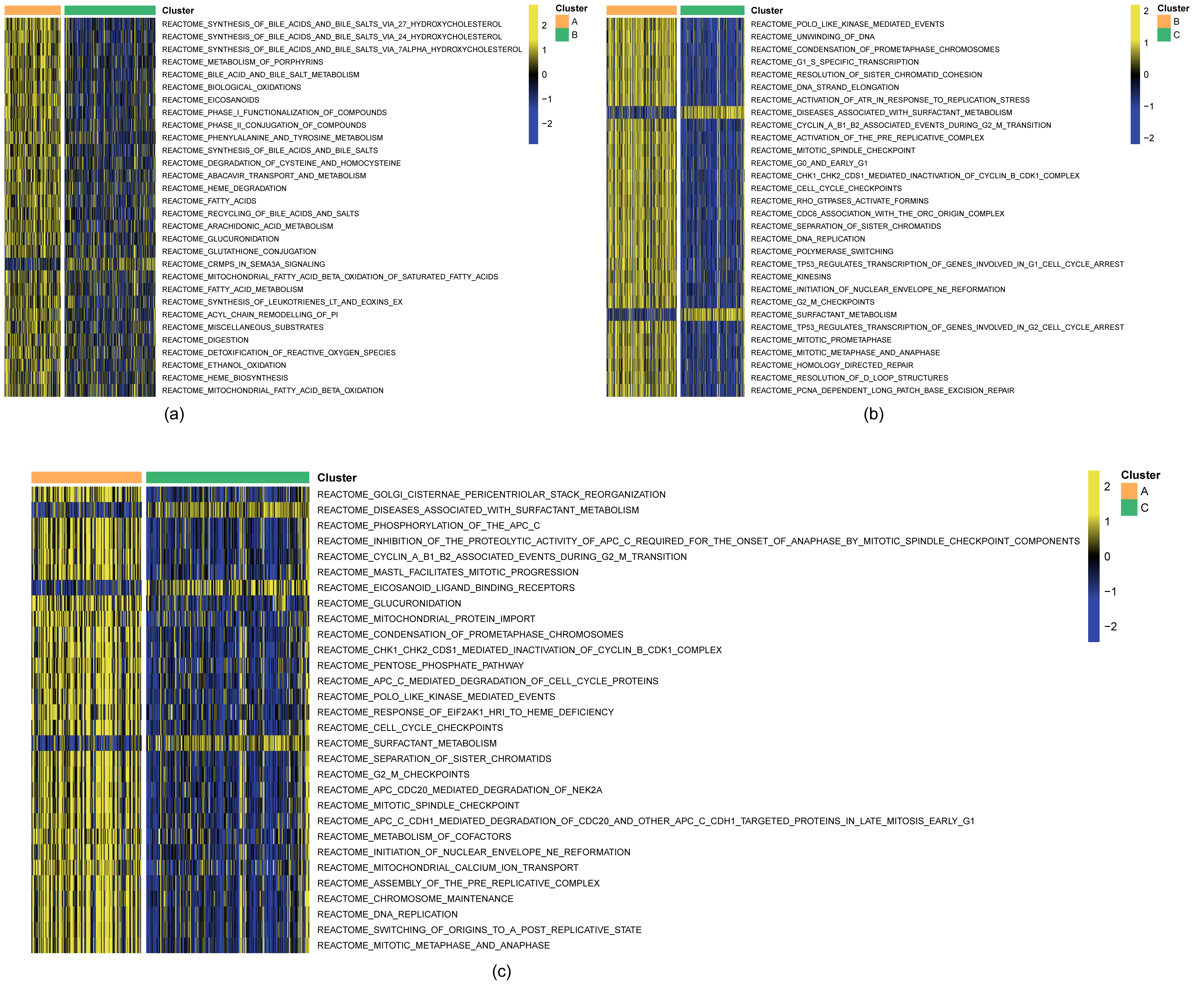

Supplement: Supplementary file 6 [file Image1.tif]

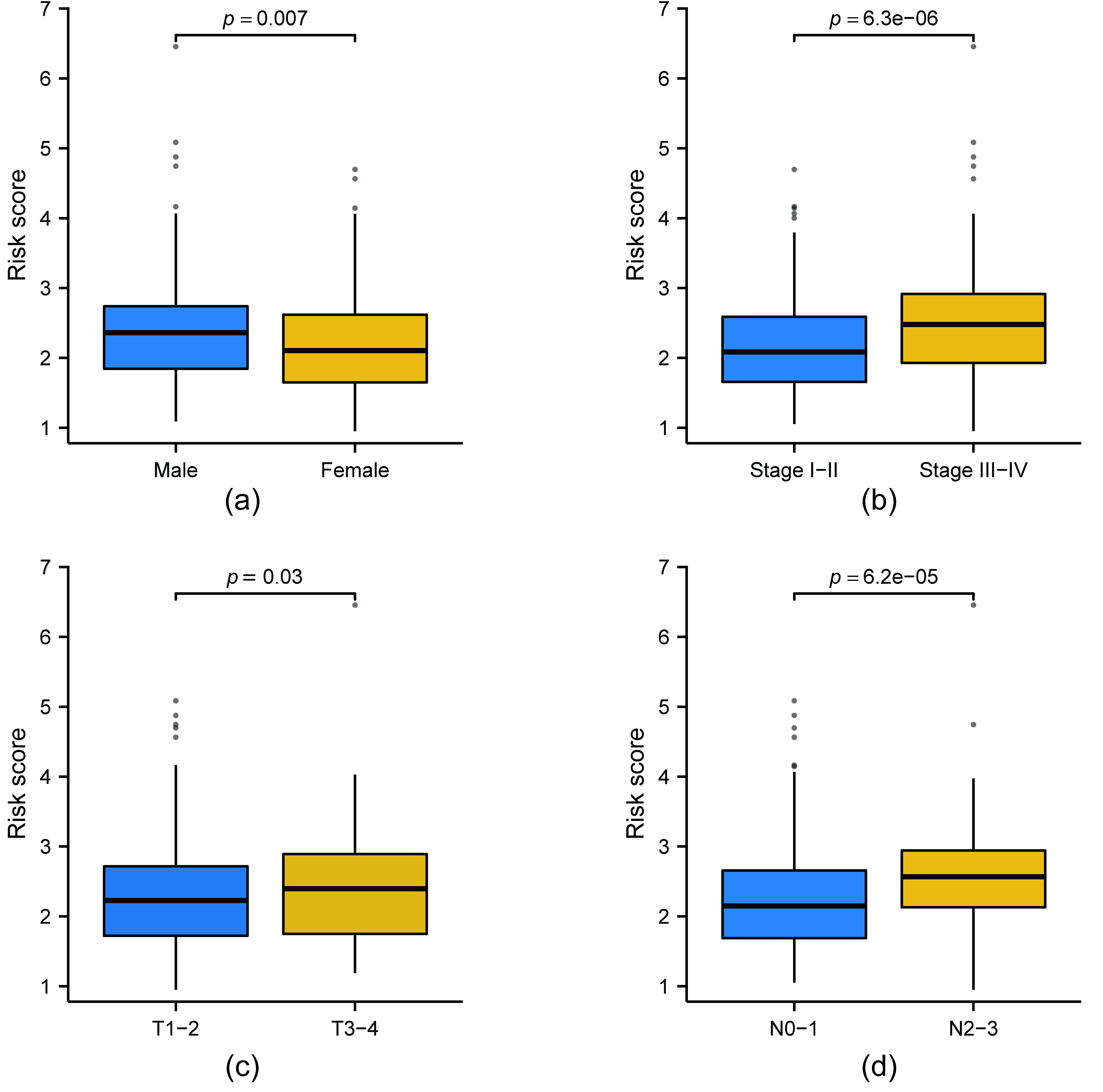

Supplement: Supplementary file 9 [file Image5.tif]
